# Managing severe yellow fever in the intensive care: lessons learnt from Brazil

**DOI:** 10.1093/jtm/taz043

**Published:** 2019-07-02

**Authors:** E G Kallas, A Wilder-Smith

**Affiliations:** 1 Department of Infectious and Parasitic Diseases, School of Medicine, University of São Paulo, São Paolo, Brazil; 2 Department of Disease Control, London School of Hygiene and Tropical Medicine, London, UK; 3 Heidelberg Institute of Global Health, University of Heidelberg, Heidelberg, Germany

Recent years have seen a resurgence of yellow fever (YF) that has also affected travellers.^1^ In 2016, Angola experienced its first major YF outbreak for decades that rapidly spread via mobility networks within the country and to neighbouring nations.^2^ At the same time, 11 unvaccinated Chinese working in Angola were infected with YF virus and exported the virus to China, the first documented exportation of YF via travellers to Asia, thereby underpinning the need for better preparedness for YF spread into Asia.^3^ In 2016, YF outbreaks also emerged at the gates of the most populated areas of metropolitan São Paulo and Rio de Janeiro in Brazil. Accordingly, the last decade also had a record number of international travellers infected by YF virus returning to Europe and the US.^4^

Clinicians in non-YF endemic countries have to be equally equipped to manage complicated YF as clinicians working in YF endemic areas, considering the high percentage of symptomatic patients who develop severe disease. Ho’s paper in this issue of the Journal of Travel Medicine presents one of the biggest cohorts of severe YF cases, thus offering many lessons for clinical management.^5^

YF can be a treacherous disease as its initial clinical presentation may be quite benign and non-specific, but can then take a fulminant course within a matter of a few days. Clinicians need to be highly suspicious of YF for any patient with an epidemiological link to potential acquisition, as earlier diagnosis may result in better management of this life-threatening disease. Upon diagnosis, the clinician may be able to identify patients with risk factors for developing more severe YF, such as those patients with older age, or presenting with high AST levels^6^ and high neutrophil counts and YF viral load.^7^ In the work by Kallas *et al*., higher levels of creatinine and bilirubin, as well as coagulopathy, were associated with higher mortality in the univariate analyses but were interestingly not independent predictors of death as determined by multivariate analyses.^7^ Ho’s paper narrows down on those with more severe clinical presentation as it describes the clinical outcomes for those patients admitted to the intensive care unit (ICU). The high frequencies of acute renal failure (84%), bleeding (66%) and pancreatitis (58%) is quite remarkable. The reported mortality of 67% was exceedingly high in a cohort that reflects the severe end of the clinical spectrum of YF. The in-hospital fatality rate was 67%, with the median interval between onset of symptoms and death being 9 days. Twenty-six (49%) died within the first 72 hours in ICU. Such high case fatality rates (CFRs) of patients requiring ICU underpin the need for novel therapeutic approaches to improve patient outcome.

Some laboratory findings were highly prevalent at ICU admission: high levels of transaminases, low platelets, greater prothrombin time, decreased factor V and fibrinogen, elevated ammonia, amylase and lipase, lower venous bicarbonate, elevated lactate, creatinine, creatinine phosphokinase and C-reactive protein.^5^

Ho *et al*. noted a high incidence of severe pancreatitis in severe YF that has not been described before to that extent.^5^ Recent descriptions of this condition in YF-deceased patients have triggered a debate whether this condition plays a role in the disease pathogenesis or is restricted to a late-stage complication of multi-organ failure.^8^

Due to severe metabolic acidosis, most patients required renal replacement therapy (73%). Continuous hemodialysis was used, and high concentrations of bicarbonate in the dialysis solution were required by the majority of patients. New therapy strategies included prophylactic anticonvulsant drugs and plasma exchange. Due to the high frequency of gastric bleeding, therapeutic doses of intravenous proton pump inhibitors were administered. Additionally, seizures occurred in 24% of patients, even without substantial intracranial hypertension. Due to the high frequency of seizures in the earlier admitted cases, it was decided to commence anticonvulsant drugs in patients with any symptoms of hepatic encephalopathy or arterial ammonia levels >70 μmol/L. Before this change in management was made, 14 of 50 (28%) patients developed seizures.^5^ After initiation of prophylactic anticonvulsant therapy, 5 of 29 (17%) patients developed seizures. Patients with diabetes mellitus had a higher CFR of 80%, while patients without diabetes had a CFR of 65%, although this difference did not reach statistical significance due to the small sample size.

In Ho’s cohort of patients with laboratory confirmed YF admitted to the intensive, three patients had received YF vaccination within the past 10 days. Viscerotropic disease was excluded as no vaccine derived virus could be isolated. These three cases reinforce the fact that onset of vaccine protection only occurs 10 days after vaccination, which is the basis for the recommendation to travel only 10 days after vaccination.

There were also three cases with breakthrough YF disease regardless of vaccination. Despite a very high vaccine efficacy, breakthrough disease is expected as no vaccine, including YF vaccine, has a 100% efficacy. However, a dearth of data exists on the risk of breakthrough disease. These three cases could either be due to true breakthrough disease, errors in vaccination records (these patients did not receive the vaccine), or disruption of cold chain requirements in the vaccine handling, storage or transportation. Stringent surveillance is needed to document breakthrough disease after YF vaccination, as such data will also help decide whether one lifetime dose is sufficient, or two doses are necessary.^9^
